# The beliefs of medical faculty students about thirdhand smoke

**DOI:** 10.18332/tid/157202

**Published:** 2023-01-31

**Authors:** İbrahim Güven Çoşğun, Şule Çilekar, Aydın Balcı, Beyza Nur Köymen, Sena Moral, Batıkan Nur, Berkay Yetim

**Affiliations:** 1 Department of Pulmonology, Afyonkarahisar Health Sciences University, Afyonkarahisar, Turkey; 2 Faculty of Medicine, Afyonkarahisar Health Sciences University, Afyonkarahisar, Turkey

**Keywords:** beliefs, medical students, tobacco, thirdhand smoke

## Abstract

**INTRODUCTION:**

Thirdhand smoke is the toxic remnant, including pollutants and by-products, of tobacco smoke that remains in the environment after the use of tobacco products. This study aimed to evaluate the relationships between the demographic characteristics and the beliefs about thirdhand smoke of medical faculty students.

**METHODS:**

This descriptive cross-sectional study was conducted with 392 medical faculty students at Afyonkarahisar Health Sciences University. All the participants completed the Beliefs About ThirdHand Smoke (BATHS) questionnaire. Demographic data such as age, gender, year of study, family income level, and place of residence were recorded and evaluated together with tobacco product usage status. Factors (demographic data such as gender) affecting the BATHS scale and sub-scales were analyzed using SPSS software.

**RESULTS:**

The study included 392 medical students. The students comprised 59.7% females and 40.3% males. The students had never used tobacco products (68.1%), 13% were previous users, and 18.9% were active users. The majority of the students stated that thirdhand smoke was harmful to the health of children (90%) and adults (85%) and that thirdhand smoke could remain in a room for days (79%). When the relationships were evaluated between the BATHS scale overall and the health and permanence subscales, and the demographic characteristics of the students, no statistically significant difference was determined according to gender, place of residence, family income level, and tobacco use status.

**CONCLUSIONS:**

This study has provided information for the first time about the beliefs of medical faculty students about thirdhand smoke, and the relationships were investigated between these beliefs and gender, place of residence, family income level, and tobacco use status. The results of the study demonstrated that the students had a strong awareness of the harm of thirdhand smoke and of environmental permanence, and these beliefs did not change according to their own tobacco use status.

## INTRODUCTION

Tobacco use is a major risk factor for cardiovascular and respiratory diseases, with over 20 different types or subtypes of cancer^1^. The prevalence of tobacco smoking in the global population is 22.3%^2^. In a recent study, the prevalence of smoking among medical students ranged from 13% to 34%^3^. Globally, tobacco kills more than 8 million people every year; 7 million of these deaths are caused by direct tobacco use, while 1.2 million deaths are due to exposure to secondhand smoke^4^. The content of tobacco smoke is rich in mutagens and carcinogens, including polycyclic aromatic hydrocarbons (PAH), hydrocarbons, nitrosamines, aromatic amines, aldehydes, and phenolic and nitrous components^5,6^. More than 60 carcinogens are found in tobacco smoke^7^. Secondhand smoke (passive smoking) comes from the passive breathing of environmental tobacco smoke and comprises the main flow and side smoke^8^. In recent years, it has come to notice that the risk of exposure to tobacco smoke is not finished when the tobacco product is extinguished but continues in the absence of tobacco products. In this context, ‘Thirdhand Smoke’ has emerged as a new concept^9^. Thirdhand smoke (THS) has recently started to attract attention because of its potentially harmful effects on those exposed to it^10^. THS has a lower concentration than secondhand smoke (passive smoking) but causes longer exposure. THS refers to the tobacco smoke pollutants and by-products in the environment after the use of tobacco products^11-14^. THS is a toxic remnant that stays in the atmosphere for a long time^15^. When tobacco is smoked, the particles within it settle on surfaces and are absorbed when on surfaces such as hair, clothes, carpets, furniture, and wall hangings.

Tobacco smoke can remain on surfaces for days or weeks^16^. It has been reported that THS can remain on material for more than 1.5 years^17^. The remnants on surfaces go through an ageing process that changes the structure and enters into a reaction with common indoor air pollutants such as nitrous acid and ozone^18-20^. As a result of the transformation of remnants on surfaces with ozone and nitrous gases, highly carcinogenic formaldehyde and tobacco-specific nitrosamines [(methylnitrosamino)-4- (3-pyridyl) butanal (NNA) and 4 (methylnitrosamino)-1-(3-pyridyl)-1-butanone (NNK)] may often emerge^21,22^. THS can be taken into the body by respiration or through the skin^23,24^. Infants and young children are more exposed to THS as they breathe more quickly, have thinner skin and spend more time in areas where dust has collected^25^.

In a study by Matt et al.^7^, THS was found to be associated with high nicotine levels in the hands of non-smokers who lived in houses where cigarettes had been previously smoked, and this led to unwanted exposure. Several studies of animal models and human cell lines have presented evidence of the harmful effects of THS^26,27^. There has also been shown to be an increase in DNA fragmentation of human cells exposed to THS^28^. It has been reported that THS can increase the risk of thrombosis-based diseases in A/J mice and can exacerbate asthma pathology^29,30^.

Quarantine precautions were implemented throughout the world during the COVID-19 pandemic. This resulted in a great increase in the time spent at home, and, therefore, greater exposure to indoor air pollutants, including the toxic substances of tobacco smoke^31^. THS is found in many enclosed spaces, including homes, public buildings, rented houses and apartments and rented cars, and despite the smoking ban can affect non-smokers. Although the restrictions in public places are promising, these restrictions have made passive smoking in home environments the main source of THS^23^. In a study related to the smoking ban, it was shown that young children could be less protected by these restrictions than adults^32^.

This finding shows that having the correct information about THS and its harmful effects can contribute to creating a smoke-free environment. However, there are still very few studies that have evaluated beliefs about THS. When it is considered that the effects of THS on health could be significant. The information about the level of the THS beliefs of medical faculty students is important, as they will be health service providers and are researchers of the future. This study aimed to evaluate the beliefs of medical faculty students about thirdhand smoke.

## METHODS

### Study design and population

This cross-sectional, analytical study was conducted with students of Afyonkarahisar Health Sciences University Medical Faculty, Turkey, who were receiving education in February 2022. An online questionnaire was sent to all medical faculty students studying. Students who answered the questionnaire were included in the study.

### Study questionnaire

The Beliefs About ThirdHand Smoke (BATHS)© scale was developed by Haardörfer et al.^33^ to determine beliefs about THS. In this study, the Turkish version of the BATHS questionnaire was used (BATHS-T), which is valid and reliable for the Turkish population^34^. The BATHS-T scale is formed of 9 items. The survey consisted of two parts: evaluation of health effects (items 1, 2, 3, 7, 8); and permanence in the environment (items 4, 5, 6, 9). Each item has a 5-point Likert-type response: 1) I definitely disagree; 2) I disagree; 3) I am undecided; 4) I agree; and 5) I definitely agree. In the original questionnaire, the Cronbach alpha value was 0.91 overall, and 0.88 for the two subscales. Similarly, the Turkish version has a Cronbach alpha value of 0.90 overall, and 0.81 for the permanence subscale, and 0.86 for the health subscale. In the current study, the Cronbach alpha values obtained were 0.922 for the general scale, 0.873 for the health subscale, and 0.844 for the permanence subscale. The Mann-Whitney U-test and the Kruskal-Wallis test were used in the group comparisons.

### Variables

The sociodemographic information of the students in the study, including age, gender, year of study, place of residence (house, university hall of residence, family home), family income level, smoking status and THS beliefs, were evaluated using an online questionnaire. The ages of the study participants were categorized to obtain close similarity to the distribution of the continuous age variable measured in the study universe (18–19, 20–21, and ≥22 years). The family income level was defined as very good, good, average, below average, and low. The accommodation of the students was classified as a private hall of residence, state hall of residence, and student house. Students were asked if they had ever smoked tobacco products. Those who had ever smoked were further asked whether they had smoked tobacco products within 30 days. Smoking status was defined as never, former (ever smoked, but not within 30 days), and current (smoked tobacco product in past 30 days).

### Statistical analysis

Statistical evaluations were performed using IBM ® SPSS statistics of Windows. Descriptive analyses were conducted to calculate the frequencies and proportions of categorical variables. The general and health and permanence subscale scores of the THS questionnaire were calculated as median and interquartile range (IQR) values. To determine differences in these scores between categorical groups, non-parametric tests were used. The Mann-Whitney U-test was applied to comparisons of the median values of two groups, and the Kruskal-Wallis test to comparisons of more than two groups. Conformity of the total points of the scale and subscales to normal distribution was evaluated with the Shapiro-Wilk test, and the data were determined not to show normal distribution. A value of p<0.05 was accepted as statistically significant.

## RESULTS

The evaluation was made of 392 medical faculty students comprising 234 (59.7%) females and 158 (40.3%) males with a mean age of 21 years (range: 18–33 years). In all, 267 (68.1%) students reported never having used tobacco products, 51 (13%) were former users, and 74 (18.9%) were active users. The active tobacco product users comprised 27 (36.5%) females and 47 (63.5%) males. The tobacco products used were packet cigarettes by 44, self-rolled cigarettes by 12, water-pipe by 12, electronic cigarettes by 4, and a tobacco heating system by 2. The highest smoking rates were seen in students in the 5th year of study (n=19; 25.7%) and in the 3rd year (n=13; 17.6%). Those who used tobacco products comprised 10 (13.5%) living in a private hall of residence, 10 (13.5%) in a state hall of residence, and 54 (73%) in a shared student house. There was reported to be a fragmented family structure by 2 (2.7%) of the students who used tobacco products, by 13 (4.9%) of those who did not use tobacco products, and by 9 (17.6%) of former users. Family members with smoking dependence were evaluated, and of the students who smoked, 12 (16.2%) reported that their mother smoked, 24 (32.4%) that their father smoked, 23 (31.1%) that one sibling smoked, 2 (2.7%) that two siblings smoked, and 2 (2.7%) that 3 siblings smoked. Of the students who smoked, 41 (55.4%) reported that their closest friend smoked. The first cigarette of the day was smoked 1 hour after waking in the morning by 55 (74.3%) students and immediately on waking by 19 (25.6%). There were significant differences in gender, type of settlement, and family income with smoking status. Smoking was higher among male students 47 (63.5%), than female 27 (36.5%) students, with a significant difference (p<0.001). Smoking was higher living in a shared student house 54 (73%) than in private halls of residence 10 (13.5%) and state hall of residence 10 (13.5%) (p=0003). Smoking was higher among students’ family income levels defined as good 26 (35.1%) and average 34 (45.9%), than other family income levels (p=0011). There was no significant difference among students’ years of study with tobacco use status.

**Figure 1:**
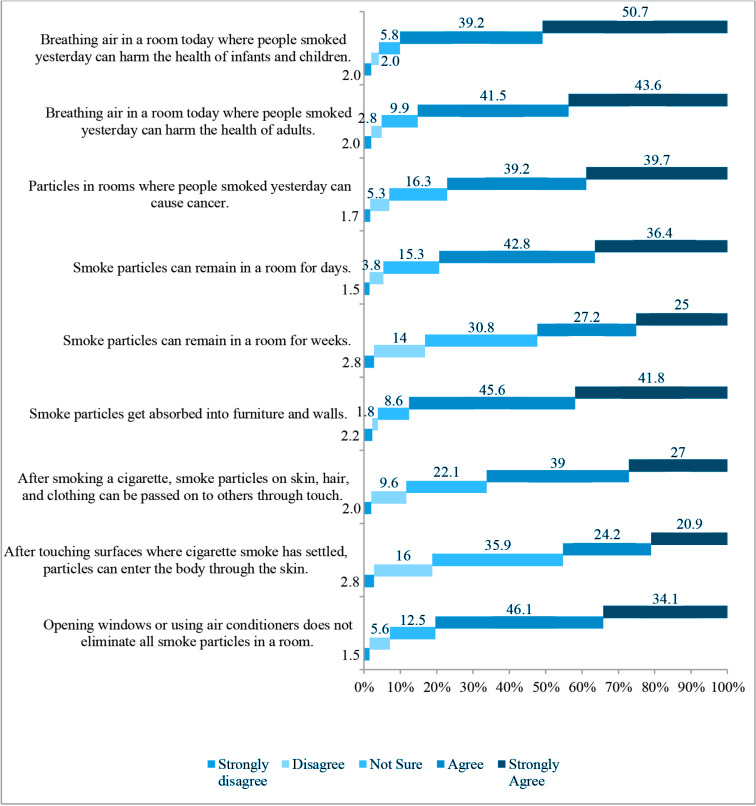
Medical student’s response to each of the 9-items assessed in the Beliefs About Thirdhand Smoke (BATHS) scale, Turkey, 2022 (N=392)

In the BATHS-T questionnaire, which evaluated the views of the students about THS, the statement, ‘breathing the air today in a room where people smoked cigarettes yesterday can be harmful to the health of infants and children’ was given responses of ‘I agree’ and ‘I definitely agree’ by 353 (90%) students (Figure 1). The vast majority of the students (79.4%) agreed and definitely agreed with the statement of ‘smoke particles can remain in a room for day’. In addition, 52.3% agreed and definitely agreed with the statement of ‘smoke particles can remain in a room for weeks’. The questionnaire item with the lowest rate of agreement (45.1%) was ‘after touching surfaces where cigarette smoke has fallen; particles can be absorbed through the skin’. When the relationships were evaluated with Mann-Whitney U-test, Kruskal-Wallis test between the BATHS-T scale overall and the health and permanence subscales and the demographic characteristics of the students, no statistically significant difference was determined between gender, place of residence, family income level, and tobacco use status (Table 1).

**Table 1:** The thirdhand tobacco smoke (THS) beliefs questionnaire scores according to the characteristics of the study participants, Turkey, 2022 (N=392)

*Characteristics*	*THS overall beliefs score*		*THS health beliefs score*		*THS persistence beliefs score*	
	*Median (IQR)*	p*	*Median (IQR)*	p*	*Median (IQR)*	p*
**Gender**						
Male	36.0 (11.0)	0.727	20.0 (6.0)	0.765	16.0 (5.25)	0.727
Female	36.0 (9.0)		20.0 (5.0)		16.0 (5.0)	
**Family income**						
Very good	32.0 (13.25)	0.191	17.5 (8.0)	0.215	14.0 (5.5)	0.188
Good	32.0 (10.0)		20.5 (5.75)		16.0 (4.75)	
Average	36.0 (9.0)		20.0 (5.0)		16.0 (4.0)	
Below average	36.0 (12.0)		21.0 (7.0)		16.0 (5.5)	
Poor	35.0 (20.25)		20.0 (11.5)		15.0 (8.75)	
**Place of residence**						
Private hall	35.0 (10.0)	0.46	20.0 (5.0)	0.649	16.0 (5.5)	0.591
State hall	36.0 (6.75)		20.0 (3.75)		16.0 (3.75)	
Shared student house	36.0 (10.0)		20.0 (6.0)		16.0 (5.0)	
**Tobacco use**						
Never smoked	36.0 (9.0)	0.431	20.0 (5.0)	0.878	16.0 (5.0)	0.212
Ever smoked (former/current)	36.0 (10.0)		20.0 (6.0)		16.0 (5.0)	

* Mann-Whitney U-test and Kruskal-Wallis test for comparisons of three or more groups.

## DISCUSSION

Considering of effect tobacco products on health, it is important to evaluate the medical students’ beliefs towards THS. Medical faculty students, as future physicians, will have a crucial role in tobacco control. In this study, it was determined how the beliefs of medical faculty students about thirdhand tobacco smoke were affected by demographic variables such as age, gender, place of residence, family income level, and the use of tobacco products. As secondhand smoke (passive smoking) is partially visible, comprehensive studies have been conducted to evaluate the negative results that have emerged. While students are informed about the negative effects of passive smoking from public announcements and stop-smoking programs, awareness of the harm of thirdhand smoke is not at that level.

With a small group of 39 participants, Escoffery et al.^35^ started the first focus group to investigate knowledge and attitudes about THS in a population with a low income. That study included open-ended questions on the subjects of THS awareness and perceived harm. It was reported that increased awareness of THS as a result of that study motivated the participants to make their homes tobacco-free.

In the current study, the majority of the participants believed that THS had a negative effect on the health of children and adults and that particles could remain on surfaces for days and weeks. Winickoff et al.^36^ reported that parents who did not use any tobacco products had stronger beliefs about the harm of THS. In a study of parents in Kuwait, those who had never used tobacco products had higher questionnaire points than former or current smokers^37^. Another study in Bangladesh reported lower levels of beliefs about THS harm in those who were currently using tobacco products and those who were former smokers^38^. In the current study, no significant difference was determined in the level of belief between smokers and non-smokers of tobacco products. Interestingly, no difference was determined in the THS belief scores according to smoking status. In the study by Haardörfer et al.^35^ in which the questionnaire was developed, two-thirds of the participants were current or former smokers of tobacco products. That no difference was determined in the current study could be attributed to the fact that two-thirds of the participants had never used tobacco products, the belief of the negative effects of tobacco product remnants was extremely high compared to other studies, and the sample was formed of medical faculty students.

In a study of parents in Shanghai, it was reported that as education level and income increased there was an increase in the THS belief scores^39^. As the sample in the current study consisted of students, the family income level was questioned, and no significant difference was determined in the THS scores of the students according to family income level.

The majority of the parents in the study by Haardörfer et al.^35^ ‘agreed’ or ‘definitely agreed’ that exposure to THS was harmful to the health of children (67.2%) and adults (60.6%). In a study by Winickoff et al.^36^ of a sample of adults living in the USA, 61.0% agreed that THS was harmful to children. Drehmer et al.^15^ formed a study group of parents living in the USA, and 91.0% agreed that exposure to THS could harm children’s health. Similarly, in the current study, 90% of the participants ‘agreed’ or ‘definitely agreed’ that THS could harm the health of infants and children.

### Strengths and limitations

Medical students as future physicians, will have an important role in tobacco control; therefore, their belief in THS behavior is of particular interest. This study provides information for the first time about the beliefs of medical faculty students about THS, and the relationships of these beliefs with gender, place of residence, family income level and smoking status were investigated. However, there were also some limitations. As the study population consisted of students from one university medical faculty only, the findings cannot be generalized to all medical faculty students or university students. Moreover, the education level, and income of the sample in this study will be different from that of a randomly selected population. Since in this study was based on self-report responses, response bias cannot be ruled out. We believe that the limitations, such as the single center, will disappear in new studies to be done. This will enable us to reveal the differences between universities by comparing them with our study and will provide new insights. The use of the standardized BATHS-T scale to evaluate the THS beliefs is a strong aspect of this study. We have assessed the place of residence, family income, smoking status, gender, years of study, family structure, family smoked status, which are important factors that may influence beliefs of THS.

## CONCLUSIONS

In this study, the tobacco usage and beliefs about THS of medical faculty students were evaluated. Our study provides novel information on beliefs about THS of medical students and how such beliefs are associated with smoking status and age, gender, the palace of residence, family income, family structure, and family smoking status. When it is considered that the effects of THS on health could be significant, the information presented here about the THS beliefs of medical faculty students is important. The medical faculty students in this study had a strong awareness of the harm of tobacco smoke and its environmental permanence; hence it can be recommended that educational messages on THS should be incorporated in the medical education/curriculum.

## Data Availability

The data supporting this research cannot be made available for privacy or other reasons.
